# Nodular Scleritis Revealing Metastasis of Breast Cancer: Diagnosis Not to Be Neglected

**DOI:** 10.1155/2020/8689463

**Published:** 2020-01-29

**Authors:** Belghmaidi Sarah, Ghazza Ahmed, Boutgayout Saloua, Hajji Ibtissam, Moutaouakil Abdeljalil, Dref Maria, Fakhri Anass, Raiss Hanane

**Affiliations:** ^1^Ophthalmology Department, Mohammed VI University Hospital, Marrakech, Morocco; ^2^Histopathology Department, Mohammed VI University Hospital, Marrakech, Morocco

## Abstract

We report the case of ocular metastasis in a 48-year-old woman presenting left eye redness and pain. The patient was followed in another health institution for nodular scleritis and received systemic corticosteroids with moderate improvement. Medical history revealed that she was diagnosed three years ago with invasive ductal carcinoma of the left breast treated by tumorectomy with ganglion dissection. An ophthalmological examination found a hard scleral nodule with vascular architectural disorganization. A biopsy was performed, and the histopathological study revealed the presence of secondary tumor proliferation of poorly differentiated carcinoma of mammary cancer. This case report shows the fact that any unusual ocular presentation, even one simulating anterior scleritis, in a patient with a history of breast cancer should raise suspicion of metastasis.

## 1. Introduction

Breast cancer is the most common neoplastic disease diagnosed in women. Metastatic tumor accounts for the most common ocular malignancy [1]. Despite recent developments in early diagnosis and active treatment, it is estimated that up to one-third of patients diagnosed with breast cancer will develop metastasis [2].

Metastasis can be found in almost any part of the eye and orbit; the incidence of ocular metastasis in breast cancer presents variable rates between 5 and 30% [3].

The most common location is the choroid; the choroid is predominantly affected with an incidence of nearly 80% of total ocular metastasis [4]. However, the scleral localization is exceptional.

The purpose of this work is to describe a case of metastatic involvement of the sclera in breast carcinoma and to discuss related literature on ocular metastasis in breast carcinoma.

## 2. Case Presentation

A 48-year-old single woman was admitted to the ophthalmic emergency department for pain and redness in the left eye. The patient was followed in another health institution for nodular scleritis and received systemic corticosteroids with moderate improvement of the symptomatology.

**Figure 1:**
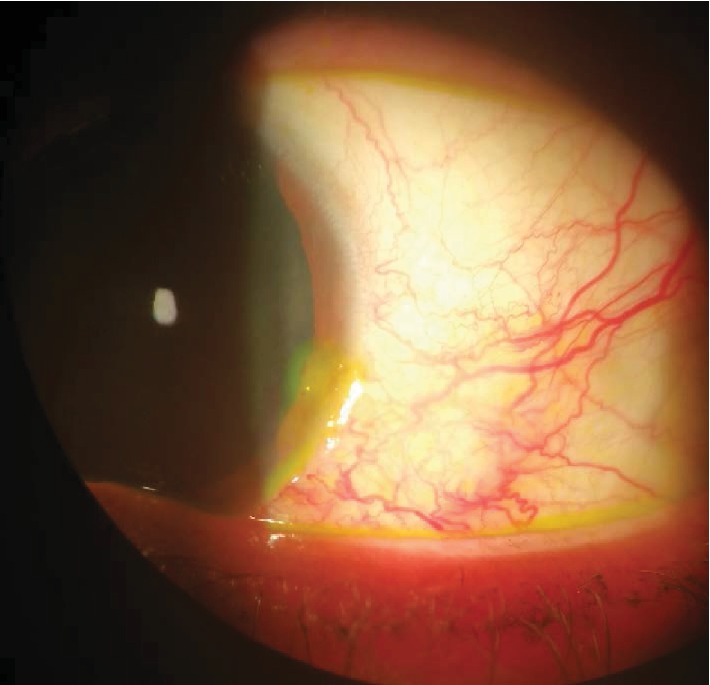
Aspect of nodular scleritis with dilatation of scleral vessels with peripheral corneal thinning.

**Figure 2:**
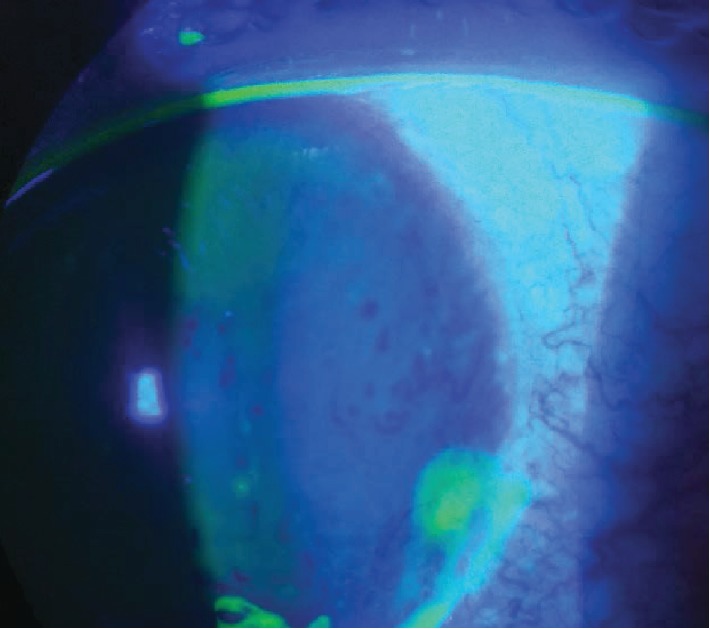
Dellen effect near the scleral nodule.

On ophthalmological examination, the best corrected visual acuity in her left eye was 0.1 LogMAR. A slit lamp examination revealed a hard scleral nodule with vascular architectural disorganization next to the nodule and dilatation of the scleral vessels (Figure 1), with a juxtanodular Dellen effect without infiltration (Figure 2). A phenylephrine test was negative. Fundus evaluation under mydriasis was unremarkable.

Medical history revealed that she was diagnosed three years ago with invasive ductal carcinoma of the left breast, and she underwent a left-side tumorectomy with ganglion dissection afterward. Postoperatively, she started treatment with adriamycin, cyclophosphamide, and docetotaxel. After the completion of 3 cycles of chemotherapy, external beam therapy of her thoracic wall was performed once a week for 8 weeks.

The clinical examination found multiple dermal cutaneous nodular lesions and an axillary lymphadenopathy of 1 cm/1 cm.

**Figure 3:**
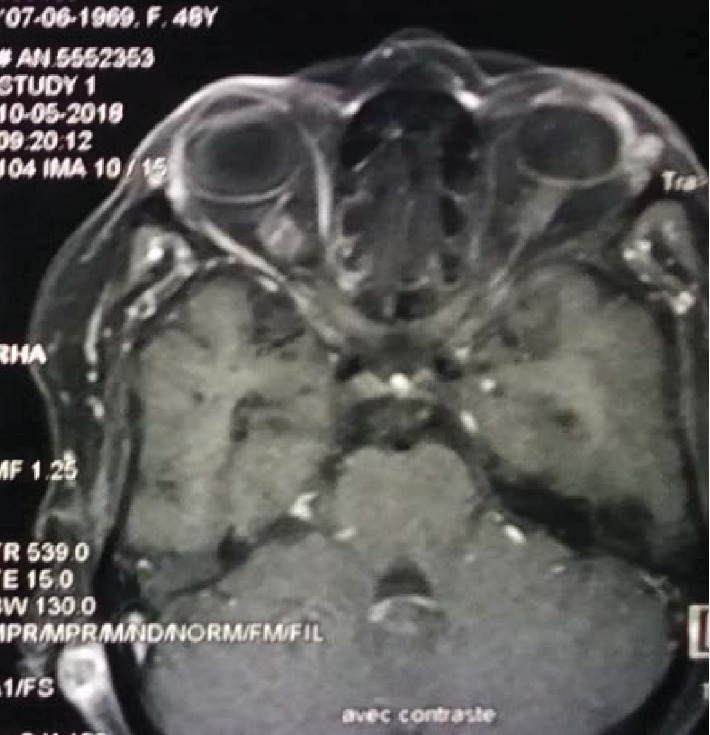
MRI objectifying a voluminous cerebellar lesion enhanced after gadolinium injection with temporal left scleral thickening.

Oculocerebral MRI showed subtentorial and supratentorial lesions: the largest of them is cerebellar and enhances after gadolinium injection, with temporal left scleral thickening (Figure 3).

Retinography with ocular ultrasound, looking for secondary choroidal localization, was performed and did not reveal abnormalities.

In addition, thoracoabdominopelvic CT showed multiple secondary-level pulmonary lesions with moderate-grade pleurisy and right intra-atrial thrombus.

Biopsy of the temporal left sclera was performed to establish a positive diagnosis and initiate appropriate treatment.

**Figure 4:**
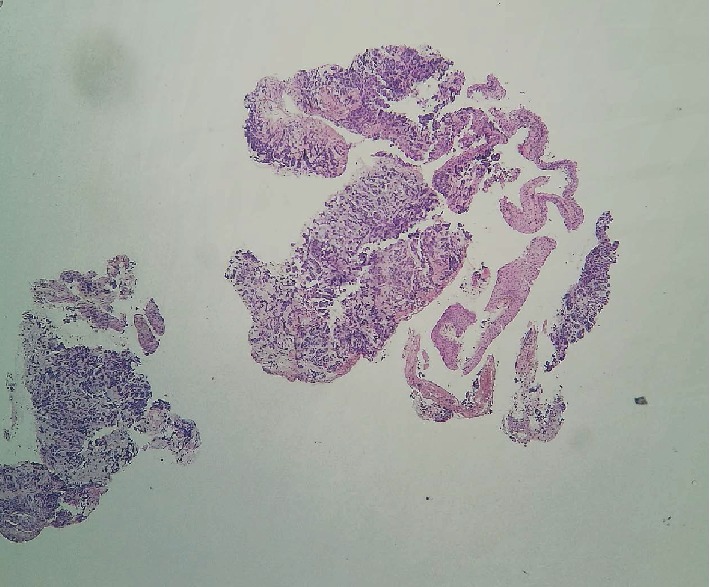
HE × 40. A conjunctival mucosa whose chorion is largely dissociated by an invasive carcinomatous proliferation.

**Figure 5:**
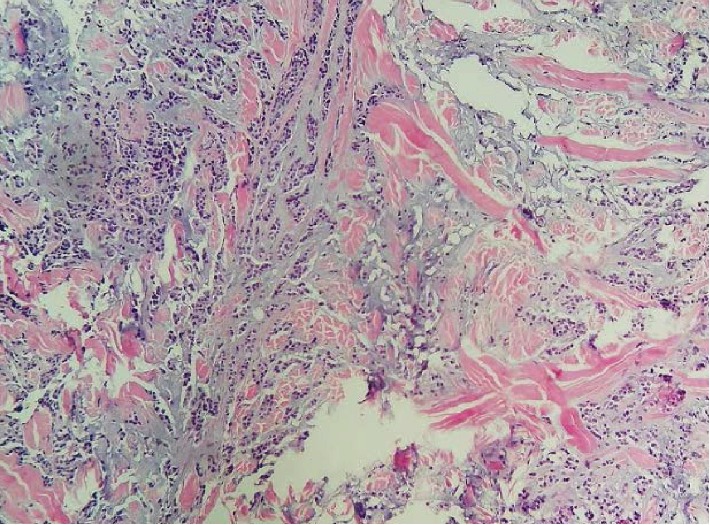
HE × 200. Carcinomatous proliferation arranged in lobules, spans, and cords. The stroma is fibroinflammatory hyalinized in places. The cells are of medium size, with anisokaryotic, hyperchromic nuclei, with irregular contours with rare mitotic figures. The eosinophilic cytoplasm is scarce.

**Figure 6:**
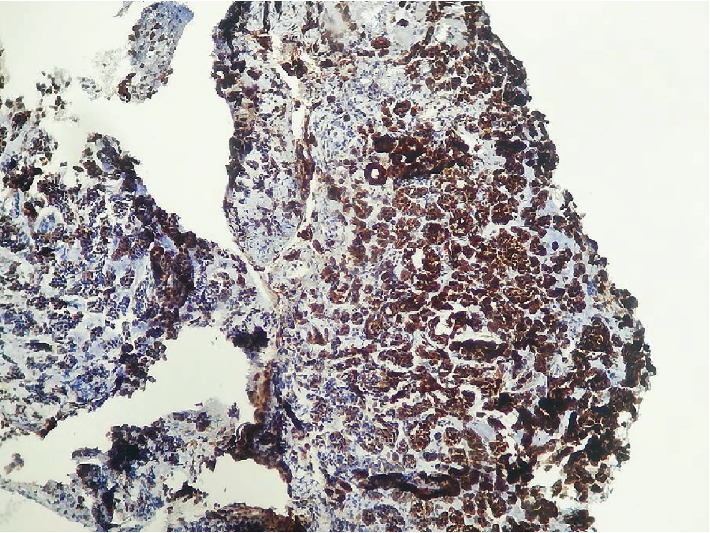
HE × 400. Intense and diffuse cytoplasmic expression of carcinomatous cells of the anti-CK7 antibody.

**Figure 7:**
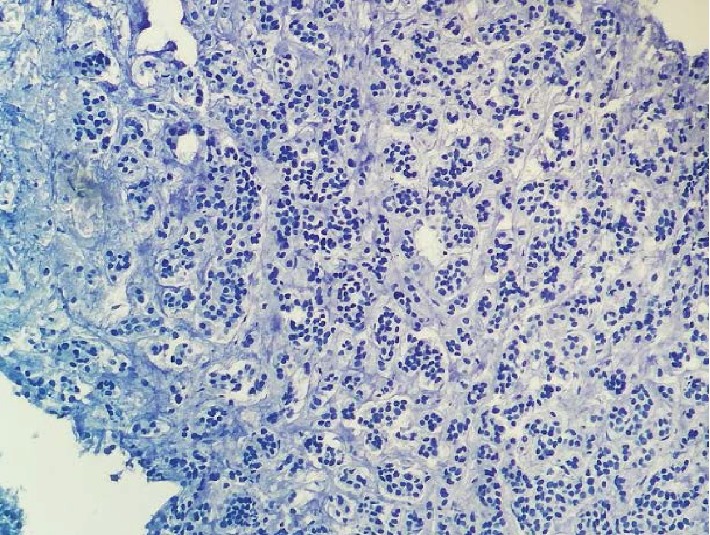
×400. Absence of expression of the carcinomatous cells of the anti-CK20 antibody.

The histopathological study revealed the presence of secondary tumor proliferation of poorly differentiated carcinoma of mammary cancer (Figures 4, 5, 6, and 7).

The final diagnosis was a scleral metastasis of breast cancer, so the patient was admitted to the oncology department for management and treatment. Unfortunately, the patient presented later respiratory distress with cardiocirculatory arrest. The resuscitation measures were in vain.

## 3. Discussion

The majority of ocular and orbital metastases are caused by breast cancer [5]. The reported incidence of breast cancer metastasis in ocular structures in clinical series varies between 8 and 10%. However, its incidence may be underestimated because of the concurrent involvement of major organs like lungs, liver, or bone which may have more serious consequences dominating the patient's clinical situation.

The ocular manifestations observed during cancers are related either to the invasion or compression of the structures of the eye or the orbit, or to a paraneoplastic syndrome secondary to autoantibodies directed against structures of the eye, or to systemic complications of cancers or their treatments [6, 7].

The uveal tissue, especially the choroid, is the primary ocular site of breast cancer metastasis accounting for 81% of total ocular metastasis [4].

Sclerotic involvement is an extremely rare form and may be revealed by a scleral nodule that may simulate anterior nodular scleritis, decreased visual acuity, ocular redness, eye pain, or watery eyes [8].

The diagnosis of an oculoorbital secondary lesion is first clinical then radiological by oculoorbital MRI in search of scleral thickening and local extension to other locoregional oculoorbital structures including invasion of the ethmoidal sinus cells or intracranial extension threatening the patient's vital prognosis in the short term (ICHT and risk of cerebral involvement).

Laboratory studies should be carried out to exclude other conditions that may cause scleral redness and thickness like granulomatous, vasculitis, endocrine, and immunologic diseases. Biopsy of the involved tissue is necessary for positive diagnosis.

Ocular metastasis usually presents in patients with breast cancer 20 to 40 months after initial diagnosis [9].

At the time of diagnosis of ocular metastasis, 85% of patients already had lung metastasis [9]. Therefore, treatment of ocular metastasis is almost always palliative.

Secondary oculoorbital lesions are of poor prognosis regardless the histological type of the primary tumor. Recent studies have shown that the 2-year survival rate was 1.3 and 27%. Another more recent study showed a mean survival of 7.4 months after the discovery of oculoorbital metastasis regardless of the histological type of the primary tumor [8].

## 4. Conclusion

Breast cancer is a common source of ocular metastasis. This case report highlights the fact that any unusual ocular presentation, even one simulating anterior scleritis, in a patient with a history of breast cancer should raise suspicion of metastasis.
